# High-Density SNP Map Construction and QTL Identification for the Apetalous Character in *Brassica napus* L.

**DOI:** 10.3389/fpls.2015.01164

**Published:** 2015-12-23

**Authors:** Xiaodong Wang, Kunjiang Yu, Hongge Li, Qi Peng, Feng Chen, Wei Zhang, Song Chen, Maolong Hu, Jiefu Zhang

**Affiliations:** ^1^Key Laboratory of Cotton and Rapeseed, Ministry of Agriculture, Institute of Industrial Crops, Jiangsu Academy of Agricultural SciencesNanjing, China; ^2^Jiangsu Collaborative Innovation Center for Modern Crop ProductionNanjing, China; ^3^Provincial Key Laboratory of Agrobiology, Jiangsu Academy of Agricultural SciencesNanjing, China

**Keywords:** *Brassica napus* L., apetalous, single nucleotide polymorphism, high-density map, quantitative trait locus, recombinant inbred line

## Abstract

The apetalous genotype is a morphological ideotype for increasing seed yield and should be of considerable agricultural use; however, only a few studies have focused on the genetic control of this trait in *Brassica napus.* In the present study, a recombinant inbred line, the AH population, containing 189 individuals was derived from a cross between an apetalous line ‘APL01’ and a normally petalled variety ‘Holly’. The *Brassica* 60 K Infinium BeadChip Array harboring 52,157 single nucleotide polymorphism (SNP) markers was used to genotype the AH individuals. A high-density genetic linkage map was constructed based on 2,755 bins involving 11,458 SNPs and 57 simple sequence repeats, and was used to identify loci associated with petalous degree (PDgr). The linkage map covered 2,027.53 cM, with an average marker interval of 0.72 cM. The AH map had good collinearity with the *B. napus* reference genome, indicating its high quality and accuracy. After phenotypic analyses across five different experiments, a total of 19 identified quantitative trait loci (QTLs) distributed across chromosomes A3, A5, A6, A9 and C8 were obtained, and these QTLs were further integrated into nine consensus QTLs by a meta-analysis. Interestingly, the major QTL *qPD.C8-2* was consistently detected in all five experiments, and *qPD.A9-2* and *qPD.C8-3* were stably expressed in four experiments. Comparative mapping between the AH map and the *B. napus* reference genome suggested that there were 328 genes underlying the confidence intervals of the three steady QTLs. Based on the Gene Ontology assignments of 52 genes to the regulation of floral development in published studies, 146 genes were considered as potential candidate genes for PDgr. The current study carried out a QTL analysis for PDgr using a high-density SNP map in *B. napus*, providing novel targets for improving seed yield. These results advanced our understanding of the genetic control of PDgr regulation in *B. napus*.

## Introduction

Oilseed rape (*Brassica napus* L., AACC, 2n = 38) is a widely planted oil crop worldwide. Rapeseed oil is not only a desirable edible oil, but is also used as a biofuel in many parts of the world (Hoekman, 2007). Additionally, oil-extracted meal from *Brassica* seeds is an excellent protein source for animal feed (Linnemann and Dijkstra, 2002). As the global demand for rapeseed products is continuously increasing, developing a high-yield variety is a main goal of *B. napus* breeding programs. An effective approach is to seek the morphological ideotype (Virk et al., 2004), and apetalous genotypes are of particular interest in breeding programs (Habekotté, 1997; Jiang and Becker, 2003).

The apetalous trait was first reported in a naturally occurring mutant of turnip (*B. campestris* L.) in *Brassica* (Ramanujam, 1940), and Buzza (1983) first detected an apetalous mutant in a spring oilseed rape. Since then, other apetalous flowers in *Brassica* species have been discovered or bred. There are several advantages to apetalous rape in yield. First and foremost, photosynthesis in cultivars without petals is more efficient, with the thick and brightly colored flowers preventing *Brassica* oilseeds from efficiently using solar energy (Jiang, 2007). The petals at the top layer of the normal flower type were reported to reflect or absorb up to 60% of incoming radiation (Mendham et al., 1981). Additionally, the apetalous cultivars have higher yield potentials than the normal type. The petal is not a photosynthesizing organ, but it consumes considerable amounts of photosynthesized assimilates during its formation and respiration (Jiang, 2007). Mendham et al. (1991) revealed that the yield of apetalous lines was higher than normal petaled cultivars. Finally, the apetalous type of rapeseeds might have a lower rate of infection from diseases distributed by petals, such as *Sclerotinia sclerotiorum*. Deciduous petals can transmit the *Sclerotinia* pathogen to healthy tissue, whereas the ascospores that land directly on the leaf surface do not germinate (Jamaux and Spire, 1999). Compared with normal petaled controls, apetalous genotypes have a much lower incidence and severity of *Sclerotinia* infection (Lefol and Morrall, 1996; Zhao and Wang, 2004). Moreover, a multitude of other diseases, such as *Botrytis cinierea* and *Peronospora parasitica*, may be distributed by petals (Lefol and Morrall, 1996). In summary, genotypes with apetalous flowers are a component of the high-yielding ideotype.

In *B. napus*, various genetic models of the apetalous trait, with different origins, are documented in many literatures. One study found that petalous flower development was controlled by one gene locus that exhibited incomplete dominance over apetalous flower development (Zhao and Wang, 2004). Most of the other studies revealed that the apetalous character in *B. napus* was regulated by recessive genes, possibly by two to four loci (Buzza, 1983; Lu and Fu, 1990; Kelly et al., 1995; Chen et al., 2006a; Zhang et al., 2007b,c). Generally, these loci independently control flower morphology; however, epistatic interactions between recessive alleles were also identified (Fray et al., 1997). In addition, the apetalous character is governed by the interaction of cytoplasmic and nuclear genes (Jiang and Becker, 2003). Although much attention has been paid to the inheritance of the apetalous character in *B. napus*, questions concerning the genetic basis remain open. Plants having less than a 10% petalous degree (PDgr) were considered apetalous (Buzza, 1983), but the distribution of PDgr in segregation generation is consecutive and should be treated as a quantitative trait (Zhang et al., 2007a). Quantitative trait loci (QTLs) mapping is a preliminary step and an effective approach to unravel the genetic architecture of complex quantitative traits and to identify QTLs for knowledge-based breeding (Mauricio, 2001). Fray et al. (1997) reported that five restriction fragment length polymorphism markers were significantly associated with one of two *stamenoid petal* loci, one each on A4 and C4. One random amplified polymorphic DNA marker that was tightly linked to a petal-controlled gene in *B. napus* was identified by Tan et al. (2003). Using a bulked segregant analysis approach, one sequence-related amplified polymorphism and one amplified fragment length polymorphism marker mapped on A4 were found to be linked to the gene controlling the petal-loss trait (Chen et al., 2006b). Based on a genetic map containing 219 markers, Zhang et al. (2007a) identified four QTLs, which were located on chromosomes A5, A6, A8, and C5, associated with the apetalous phenotype.

High-density maps could increase the precision of QTL localization and the estimation of QTL effects in biparental populations (Stange et al., 2013). Since the first molecular linkage map in *B. napus* was reported by Landry et al. (1991), various types of populations have been constructed for mapping QTLs associated with seed oil content, seed fatty acid concentrations, flowering time, seed yield and yield-related traits (Qiu et al., 2006; Quijada et al., 2006; Udall et al., 2006; Long et al., 2007; Basunanda et al., 2010; Chen et al., 2010; Cai et al., 2012; Ding et al., 2012; Wang et al., 2013; Wang et al., 2015a). However, most of these genetic linkage maps were constructed based on PCR markers with low densities. Single nucleotide polymorphisms (SNPs) are the most frequent polymorphism in the genomes of crops (Vignal et al., 2002), and they have been widely used in rice (Huang et al., 2010), wheat (Mochida et al., 2003), and maize (Ching et al., 2002). In *B. napus*, SNPs were also used for high-density genetic map construction and the fine mapping of important genes. Delourme et al. (2013) developed an integrated genetic map, which was comprised of 5,764 SNPs and 1,603 PCR markers, with a genetic length of 2,250 cM. Based on the 6 K SNP array harboring 5,306 probes for *B. napus*, Raman et al. (2014) constructed a genetic linkage map covered 2,514.8 cM, including 613 SNPs and 228 non-SNPs, and Cai et al. (2014) constructed a genetic map containing 2,115 markers (1,667 SNPs and 448 SSRs), with a length of 2,477.4 cM. Chen et al. (2013) constructed a SNP bin map containing 8,780 SNP loci and a presence/absence variation map containing 12,423 dominant loci. In 2012, the *Brassica* 60 K SNP BeadChip Array comprised of 52,157 SNP loci was produced (Snowdon and Iniguez Luy, 2012; Edwards et al., 2013), which was developed by an international consortium using preferentially single-locus SNPs contributed from genomic and transcriptomic sequencing in genetically diverse *Brassica* germplasm (Liu et al., 2013). This paved the way for the high-throughput and cost-effective construction of a high-density genetic *B. napus* map. Using the 60 K SNP BeadChip Array, Liu et al. (2013) constructed a linkage map containing 9,164 SNP markers covering 1,832.9 cM, and mapped the major QTL for seed color corresponding to a physical region of 620 kbp. Zhang et al. (2014) constructed a map covering a length of 2,139.5 cM with average distance of 1.6 cM between adjacent markers. Nevertheless, to the best of our knowledge, a QTL analysis for the apetalous trait using high-density SNP genetic linkage map in *B. napus* has not been performed.

The objectives of the present study were to: (1) construct a high-density genetic map using the *Brassica* 60 K Infinium SNP array and SSRs; and (2) investigate the QTLs for PDgr in *B. napus* across five experiments. The results will provide information useful for understanding the genetic control of apetalous in *B. napus*, and the major QTLs will lay a foundation for use in breeding programs to develop a variety with agronomic traits of interest for rapeseed production.

## Materials and Methods

### Plant Materials

The *B. napus* segregating recombinant inbred line (RIL) population used in this study was derived from a cross between ‘APL01’ and ‘Holly’ using the single seed descent method. The parent ‘APL01’ is an apetalous line, developed at the Institute of Industrial Crops, Jiangsu Academy of Agricultural Sciences, Nanjing, China. ‘APL01’ was selected from F_6_ generation of crosses between apetalous (Apeatlous No. 1) and normal petalous (Zhongshuang No. 4) rapeseed in 1998 (Zhang et al., 2002). ‘Apeatlous No. 1’ was bred from F_8_ generation of crosses between China rapeseed cultivar with smaller petals (SP103) and *B. rapa* variety with lower petals (LP153). ‘Zhongshuang No. 4’ was developed at Oil Crops Research Institute of the Chinese Academy of Agricultural Sciences, Wuhan, China. Except for PDgr, other agronomic traits in ‘APL01’ are quite normal. At early flowering stage, ‘APL01’ is absolutely apetalous, however, there may be one cripple petal only in a few flowers at late flowering stage. The genotype ‘Holly’ is a normally and completely petaled variety. The two parents showed the similar flowering time, which recorded from the sowing day to the day when the first flower had opened on half of the plants in the plot. A total of 550 F_9_ RIL lines were developed in 2014, and then a subset of 189 lines was randomly selected to compose the mapping population for the genetic linkage map construction. These were named the AH RIL population.

### Field Trials and PDgr Measurements

The AH population, together with the two parents, was tested in five experiments. The materials were planted in a winter rapeseed area, Dali of Shaanxi Province (coded DL), in northwest China for one year (September-May of 2014–2015), and a semi-winter rapeseed area, Nanjing of Jiangsu Province (coded NJ), in eastern China for four years (September-May of 2011–2012, 2012–2013, 2013–2014, and 2014–2015). Year-location combinations were treated as experiments, for example, 14NJ indicates the experiment was conducted during 2014–2015 at the Nanjing location. The field experiments were conducted in a randomized complete block design with two replications in both NJ and DL. The experimental unit was a two-row plot with 20 plants per row and 40 cm between the rows. The field management followed the common agricultural practices.

At least five representational plants of each plot were selected to measure PDgr during the flowering stage. The percentage of petals of an individual plant was determined by counting the number of petals on the first 25 flowers to open (Buzza, 1983). The PDgr was calculated by the following formula as described by Buzza (1983):

$$PDgr(\%)=(\overset{n}{\underset{i=1}{\Sigma }}Pi/4n)\times 100\%,$$

in which *P* represents the number of petals for each flower that was counted, with a range of numerical value of 0–4, and *n* is the total number of flowers we investigated, *n* ≥ 50.The average of the PDgr in each RIL line was used as raw data in the analysis.

### Statistical Analysis

Basic statistical analyses of PDgr were performed using SPSS 18.0 software (SPSS Inc., Chicago, IL, USA). The software package SEA-G3DH, with the mixed major gene and polygene inheritance model, was used to analyze the inheritance of the PDgr character in the AH population (Cao et al., 2013). The best fitting model was selected from 39 different models that were included in the software package according to Wang et al. (2015b). Because the PDgr was expressed as a percentage and did not fit the normal distribution model, all data were subjected to an arcsine transformation for the genetic model analysis. Genetic parameters were estimated using the best fitting model with the default settings in the software.

### SNP and SSR Marker Analysis

The genotypes of the AH RIL population and two parental lines were analyzed using the *Brassica* 60 K SNP BeadChip Array, which successfully assays 52,157 Infinium Type II SNP loci in *B. napus*. This array was developed by the international *Brassica* SNP consortium in cooperation with Illumina Inc. San Diego, CA, USA. DNA sample preparation, hybridization to the BeadChip, washing, primer extension and staining were strictly carried out according to the Infinium HD Assay Ultra manual. Imaging of the arrays was performed using an Illumina HiSCAN scanner. Allele calling for each locus was performed using the GenomeStudio genotyping software v2011 (Illumina, Inc.). SNP markers used the names that were assigned by GenomeStudio, such as “Bn-A01-p25032772”. SSR primer pairs prefixed “CB” and “BRAS” were published by Piquemal et al. (2005); “Na”, “Ol” and “Ra” were developed by Lowe et al. (2004); “MR” were published by Uzunova and Ecke (1999) and “BnGMS” were developed by Cheng et al. (2009).

### Construction of the Genetic Linkage Map and Alignment of the *B. napus* Reference Genome

All SNPs that were polymorphic between ‘APL01’ and ‘Holly’, as well as having less than 5% missing data, were used for the genetic linkage map construction. Of the 52,157 SNPs in the array, 17,414 SNPs met the above requirements and were selected for further analysis. SNP marker pairs with no recombination were classified into one genetic bin (one bin corresponded to all of the markers having the same genotype scoring data) using a Perl script. Then, the selected 17,414 SNPs were grouped into 3,422 SNP-bins, containing 1 to 2,079 SNPs in each bin. Combined with 81 polymorphic SSR markers between the two parents, 3,503 loci (3,422 SNP-bins and 81 SSRs) were subsequently applied in map construction using JoinMap software Version 4.0 (Van Ooijen, 2006). Centimorgan (cM) distances were calculated by the Kosambi function for map distance (Kosambi, 1943). Markers with a mean chi-square value ≥ 3.0 were excluded in all genetic groups to ensure the high quality of the map (Wang et al., 2013).

In addition, the probe sequences of the SNPs that assigned the A and C sub-genomes of *B. napus*, were queried using the BLAST algorithm against the *B. napus* reference genome sequence to locate chromosomal positions with highly stringent parameters (*E* value ≤ 1e-10) (Chalhoub et al., 2014). Alignments between the SNP bin map and the *B. napus* reference genome were used to validate the quality of the genetic map. If a locus was mapped to multiple paralogous positions in the *B. napus* reference genome, only the location that corresponded to the particular linkage group of the locus was selected for the collinearity analysis.

### QTL Detection and Meta-Analysis

The software Windows QTL Cartographer 2.5 with a composite interval mapping model was used to estimate putative QTLs with additive effects (Zeng, 1994; Wang et al., 2007). The walking speed was set to 2 cM, and a window size of 10 cM with five background cofactors was used. The LOD threshold (2.8–3.1) for detection of significant QTLs was set by a 1,000-permutation test based upon a 5% experiment-wise error rate, and these QTLs were termed ‘identified QTLs’. Identified QTLs that were detected in different experiments with overlapping confidence intervals (CIs), may have been one single QTL. Then, identified QTLs were integrated into consensus QTLs using a meta-analysis method with the BioMercator V4.2 program (Arcade et al., 2004). If an identified QTL had no overlapping CIs with others, then it was also regarded as a consensus QTL (Wang et al., 2013).

The identified and consensus QTL nomenclature was based on the descriptions of McCouch et al. (1997) with minor modifications. For identified QTLs, a designation begins with the abbreviation “*iq*” (identified QTL), follow by the experiment and linkage group (A1–A10, C1–C9). If there was more than one identified QTL obtained in a linkage group, a serial number was added. For example, *iq12NJ.A9-2* indicates the second identified QTL for PDgr on A9 in the 12NJ experiment. For consensus QTLs, a designation begins with the abbreviation “*qPD*” (*q*, QTL; *PD*, petalous degree). For example, QTL *qPD.A9-2* indicates the second consensus QTL for PDgr on the A9 linkage group. In addition, candidate genes were identified by comparative mapping between the AH map and the *B. napus* reference genome based on the probe sequence of the SNPs. If the physical positions of aligned genes fell into the CI of a consensus QTL, then the orthologous candidate genes were assumed to be associated with the target QTL (Ding et al., 2012).

## Results

### Phenotypic Variation and Genetic Analysis for PDgr

**FIGURE 1:**
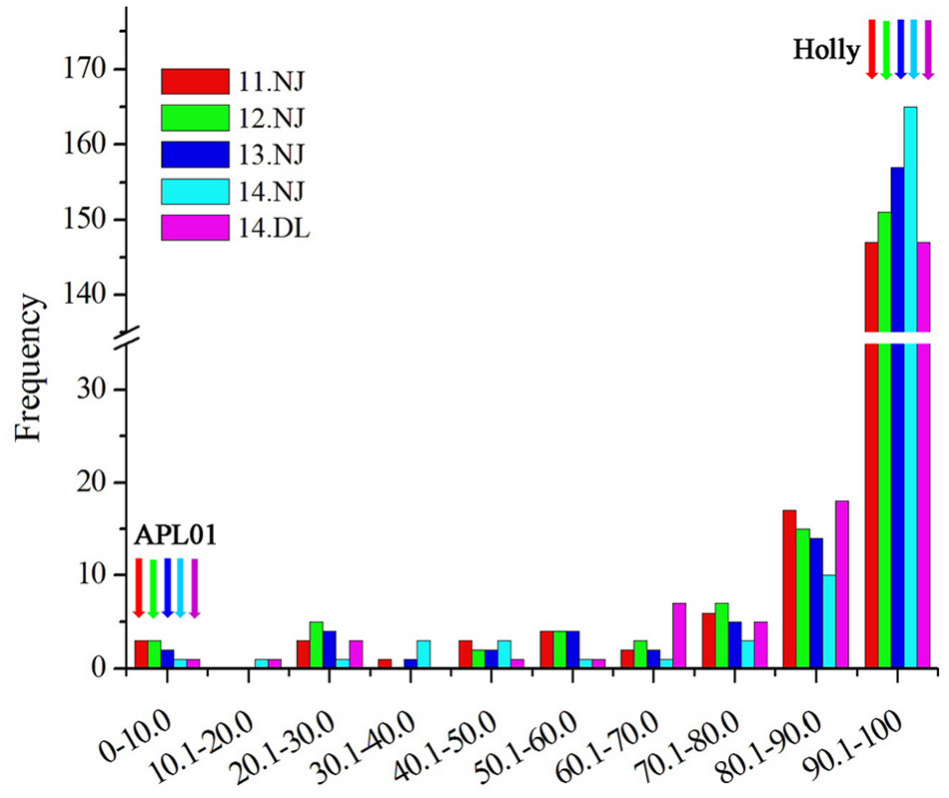
**Distribution of the petalous degree (PDgr) of the AH RIL population in five experiments.** The units of the *x*-axis are the percentage of the PDgr, and the units of the *y*-axis are the number of lines. PDgr in different experiments was discriminated using different colored boxes (11NJ, red; 12NJ, green; 13NJ blue; 14NJ, cyan; 14DL, magenta). The color arrows represent the location of the parental lines.

‘APL01’ and ‘Holly’ are the two parental lines whose repeatability is very good in the five experiments. The mean values ± SE were 0.02% ± 0.03 and 99.99% ± 0.01 for ‘APL01’ and ‘Holly’, respectively. There was a wide range of variations and consecutive distributions in the AH population (**Figure 1**), suggesting that PDgr was governed by multiple genes. However, the phenotypic values did not fit the normal distribution in the majority of the RIL lines, mostly petalled, indicating that PDgr might be determined mainly by the major genes in *B. napus*.

The mixed major gene and polygene inheritance model has been used to analyze the two parents and AH population for PDgr in the 11NJ and 12NJ experiments (Li et al., 2014). The PDgr of the AH population was controlled by the two additive major genes and the additive polygene model (MX2-Additive-A model) (Li et al., 2014). Using the same method, the best fitness genetic models for PDgr in 13NJ, 14NJ, and 14DL were analyzed in the present study. The MX2-Additive-A model was also the best fitness genetic model in the 13NJ and 14NJ experiments. However, the genetic model MX2-EA-A, which mixed two equal additive major genes with the additive polygene model, was the most suitable model for the 14DL experiment. The heritabilities of major genes ranged from 68.52% to 88.01% in the five experiments (**Table 1**), significantly higher than that of the polygenes (11.99–31.48%), indicating that PDgr in *B. napus* was determined by the combination of major genes and polygenes, but mainly by the major genes.

**Table 1:** Genetic parameters estimated in the MX2-Additive-A or MX2-EA-A model in the AH population.

First order	Experiments^b^					Second order	Experiments				
			
Parameter^a^	11NJ	12NJ	13NJ	14NJ	14DL	parameter^c^	11NJ	12NJ	13NJ	14NJ	14DL
*d(da)*	-8.24	-9.31	-9.25	-10.85	-18.41	σ^2^_p_	332.53	235.52	333.76	286.01	185.5
*Db*					-18.41	σ^2^_mg_	287.11	198.41	290.57	251.65	127.1
*I*	-21.17	-20.9	-20.44	-20.35		σ^2^_pg_	44.87	36.56	43.18	34.29	58.38
[*d*]	-28.14	-26.03	-26.48	-23.3	0	σ^2^_e_	0.55	0.56	0.01	0.07	0.02
						*h*^2^_mg_(%)	86.41	84.34	87.06	88.01	68.52
						*h*^2^_pg_(%)	13.51	15.54	12.94	11.99	31.48


*DL, Dali; NJ, Nanjing; 11, 12, 13 and 14 indicate the years 2011, 2012, 2013, and 2014, respectively. ^a^*d*: The additive effect of a major gene; *i*: The epistasis effect of a major gene; [*d*]: The additive effect of polygene. ^b^The best fitness genetic model in 11NJ, 12NJ, 13NJ and 14NJ experiments is the MX2-Additive-A model, while the MX2-EA-A model was the best fitness genetic model in the 14DL experiment. Genetic parameters estimated in 11NJ and 12NJ were reported by Li et al. (2014). ^c^σ^2^_p_, Population variance; σ^2^_*mg*_, Major gene variance; σ^*2*^_*pg*_, Polygene variance; σ^*2*^_*e*_, Environmental variance; *h*^*2*^_*mg*_ (%), Heritability of the major genes; *h*^*2*^_*pg*_ (%), Heritability of the polygene.*

### High-Density SNP Map Construction

**FIGURE 2:**
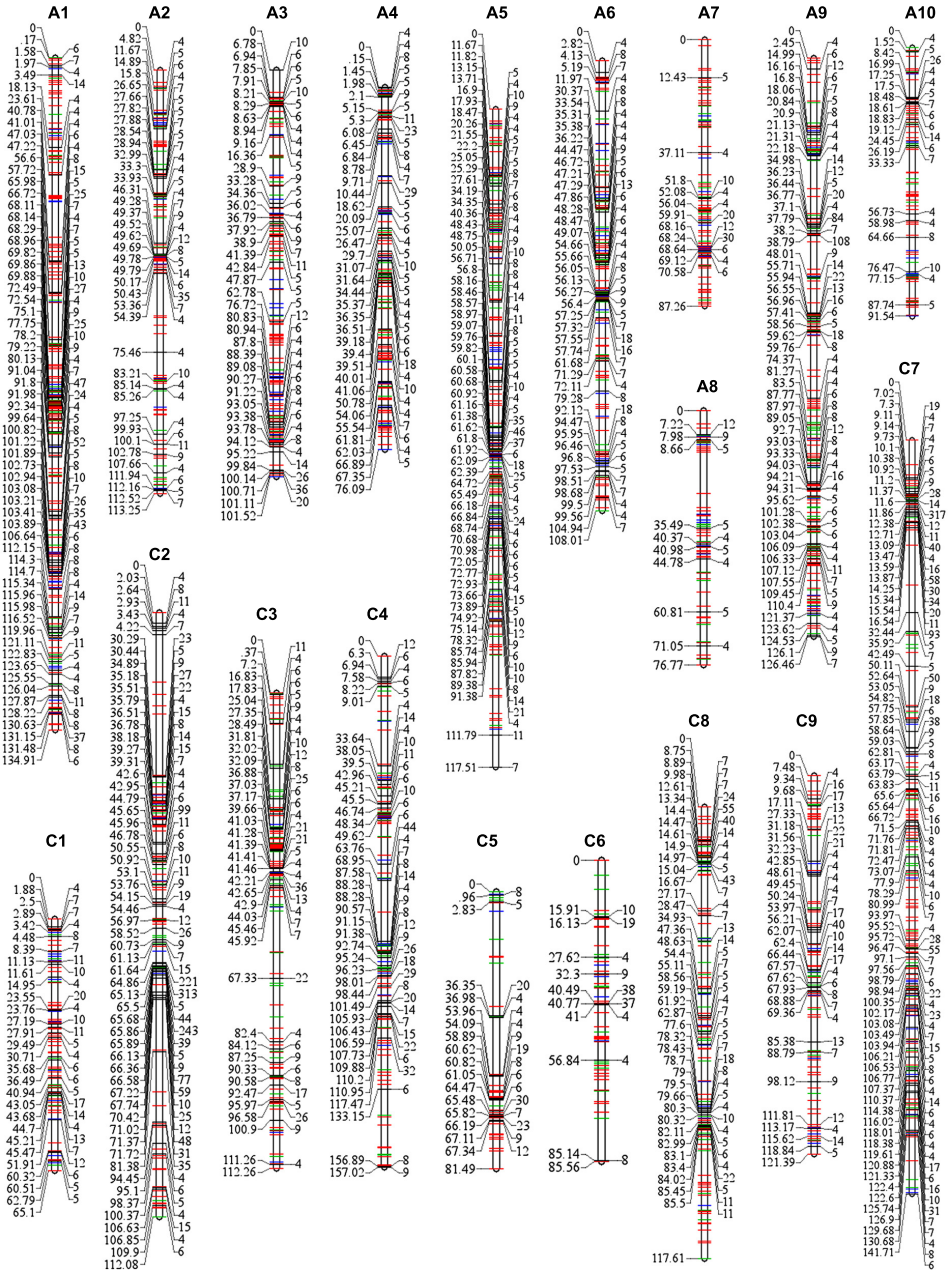
**SNPs distribution in each linkage group of the map constructed using 189 RIL individuals.** The 19 linkage groups are represented by vertical bars, designated as A1–A10 in the A sub-genome and C1–C9 in the C sub-genome. The number of SNPs in each bin is listed on the right side of the linkage groups, while the positions of the bins are shown on the left side of the linkage groups. Bins with less than three SNPs were represented by colored lines (red lines, bins involve one SNP; green lines, two SNPs; and blue lines, three SNPs), for simplicity the numbers and positions are not shown. Full details are provided in Supplementary Table S1.

Among the 3,503 loci (3,422 SNP-bins and 81 SSRs) that were used for map construction, 2,755 SNP-bins and 57 SSRs were assigned to 19 linkage groups, including 1,686 loci in the A sub-genome (A1–A10) and 1,126 in C sub-genome (C1–C9; **Table 2**, Supplementary Table S1). The number of SNP markers varied considerably across the different bins, ranging from 1 to 317, and 11,458 SNPs involved in the 2,812 loci were assigned to the genetic map (**Table 2**; **Figure 2**). A8 has the lowest number of SNPs with only 139 SNPs spanning 76.77 cM, and C2 has the most SNPs with 1,788 SNPs spanning 112.08 cM (**Figure 2**).

**Table 2:** Summary of the high-density SNP map based on the AH RIL population.

Chromosome	SNP-bins	SNPs	SSRs	SNPs+SSRs	Bins+SSRs	Length (cM)	Density	No. of loci assigned to *B. napus* genome	Genome coverage (Mb)	Total length of *B. napus* genome (Mb)	% of genome coverage
A1	270	1008	7	1015	277	134.91	0.49	210	0.20-23.24 (23.04)	23.27	99.01
A2	151	430	0	430	151	113.25	0.75	126	0.06-24.79 (24.73)	24.79	99.76
A3	184	528	2	530	186	101.52	0.55	171	2.12-28.99 (26.87)	29.77	90.26
A4	141	413	1	414	142	76.09	0.54	119	0.74-19.08 (18.34)	19.15	95.77
A5	226	836	5	841	231	117.51	0.51	202	0.09-21.62 (21.53)	23.07	93.32
A6	175	493	4	497	179	108.01	0.60	148	1.21-23.84 (22.63)	24.40	92.75
A7	118	260	3	263	121	87.26	0.72	100	1.79-22.13 (20.34)	24.01	84.71
A8	68	139	4	143	72	76.77	1.07	65	0.73-18.86 (18.13)	18.96	95.62
A9	212	832	2	834	214	126.46	0.59	172	4.14-33.83 (29.69)	33.87	87.66
A10	111	269	2	271	113	91.54	0.81	105	0.26-17.37 (17.11)	17.40	98.33
C1	100	326	2	328	102	65.10	0.64	87	0.08-13.07 (12.99)	38.83	33.45
C2	146	1788	0	1788	146	112.08	0.77	116	0.52-45.79 (45.27)	46.22	97.94
C3	136	499	2	501	138	112.26	0.81	127	0.002-26.47 (26.47)	60.57	43.70
C4	124	585	5	590	129	157.02	1.22	104	0.002-48.79 (48.79)	48.93	99.71
C5	46	215	2	217	48	81.49	1.70	40	0.15-14.91 (14.76)	43.19	34.17
C6	63	212	1	213	64	85.56	1.34	50	2.86-37.19 (34.33)	37.23	92.21
C7	229	1612	8	1620	237	141.71	0.60	199	0.63-44.32 (43.69)	44.77	97.59
C8	153	587	2	589	155	117.61	0.76	136	0.80-37.98 (37.18)	38.48	96.62
C9	102	426	5	431	107	121.39	1.13	72	1.43-48.40 (46.97)	48.51	96.83
A subgenome	1656	5208	30	5238	1686	1033.31	0.61	1418	222.41	238.69	93.72
C subgenome	1099	6250	27	6277	1126	994.22	0.88	932	310.45	406.73	76.91
Whole Genome	2755	11458	57	11515	2812	2027.53	0.72	2350	532.86	645.42	85.76


*The total length of the *B. napus* genome was reported by Chalhoub et al. (2014; http://www.genoscope.cns.fr/brassicanapus/).*

The high-density map had a total length of 2,027.53 cM with an average marker interval of 0.72 cM, covering 1,033.31 and 994.22 cM of the A and C sub-genomes, respectively (**Table 2**). The average linkage group lengths of the A and C sub-genomes were similar at 103.3 and 110.5 cM, respectively. However, the lengths of each group showed great differences, ranging from 76.09 (A4) to 134.91 cM (A1) in the A sub-genome and 65.10 (C1) to 157.02 cM (C4) in the C sub-genome. In addition, no chromosome in the genetic map displayed gaps of more than 20 cM, while C5 showed the largest gap of 16.70 cM between Bn-scaff_16082_1-p33791 and Bn-scaff_15712_10-p52253 (Supplementary Table S1).

### Alignment of the SNP Linkage Map to the *B. napus* Reference Genome

**FIGURE 3:**
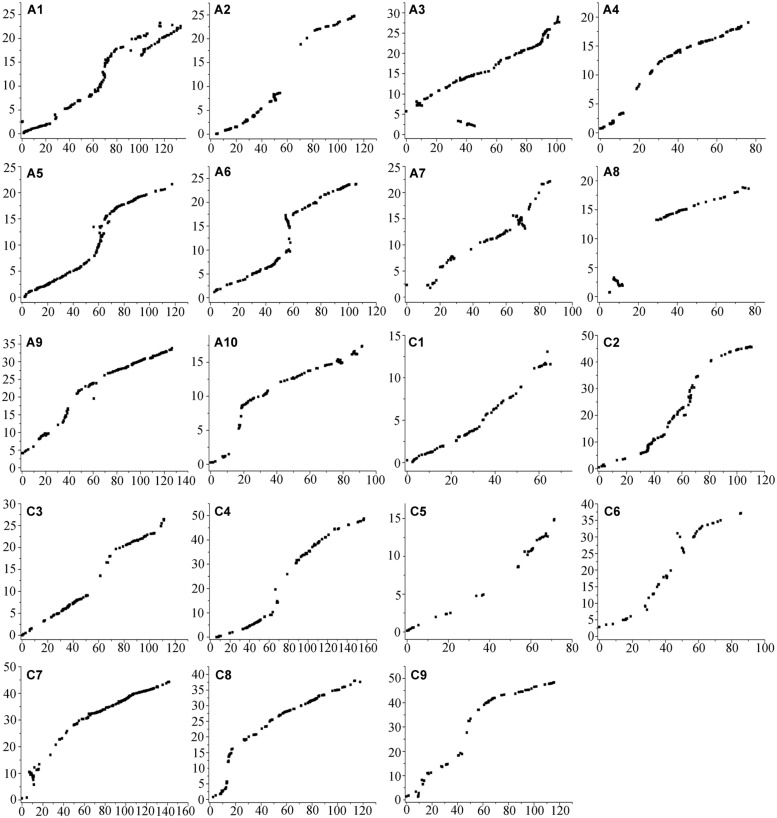
**Alignments between the SNP linkage map and the *Brassica napus* reference genome sequence.** The *x*-axis represents the genetic distance in centimorgan (cM) of each linkage group in the AH map constructed in the present study. The units of the *y*-axis are the physical distances (Mb) based on the *B. napus* reference genome sequence.

The probe sequences of all 2,755 SNP-bins that mapped to the 19 linkage groups were aligned to the *B. napus* reference genome to validate the genetic linkage maps (**Figure 3**). The results showed that 2,350 loci produced successful BLAST hits in the *B. napus* database, accounting for 85.30% of the 2,755 SNP-bins (85.63% for the A sub-genome and 84.80% for the C sub-genome).

Alignments indicated that the linkage map constructed in the present study had good collinearity with the *B. napus* reference genome sequence (**Figure 3**), suggesting the high quality of the AH RIL map. However, several inconsistencies on A1, A3, A7, and A8 were detected between the map and the *B. napus* reference genome. For example, a large inconsistency involving 80 SNP-bins and spanning a region from 100.82 to 133.75 cM (16.27 Mb of physical region) on A1 might be caused by the existence of paralogous sequences. The inconsistency from the 34.36 to 45.37 cM on the A3 chromosome, which includes 38 SNP-bins and corresponds to ∼1.23 Mb of the physical interval, may have been caused by the presence of partial homologous sequences or fragment duplications. In addition, an inversion including 41 loci and spanning from 64.33 to 71.49 cM (2.49 Mb) was identified on the A7 chromosome, and an inversion with 20 loci from 4.09 to 12.40 cM (2.54 Mb) was also identified on A8.

The total genome size of *B. napus* is estimated to be 1,130 Mb, and ∼ 645.4 Mb of the genome assembly was collectively comprised by the scaffolds (Chalhoub et al., 2014). The A sub-genome of the AH map showed good coverage of the reference *B. napus* genome, representing 93.72% of the genome assembly length, while the C sub-genome showed a much lower coverage of 76.91% (**Table 2**). The main reason was that three linkage groups, C1, C3, and C5, only represent 33.45, 43.70, and 34.17%, respectively, of the corresponding chromosomes (**Table 2**; **Figure 3**). The remaining six linkage groups of the C sub-genome account for 92.21to 99.71% of the genome assembly length.

### QTL Detection and Meta-Analysis for PDgr

Phenotypic data of PDgr in the AH population were obtained from the five different environments. QTLs for PDgr were analyzed based on the high-density SNP map, and then identified QTLs were integrated into consensus QTLs. Detailed information on identified and consensus QTLs are summarized in **Table 3.**

**Table 3:** A list of nine consensus QTLs for petalous degree obtained after the meta-analysis of 19 identified QTLs in five environments.

Consensus QTLs				Identified QTLs							
	
QTL	Peak	Marker^a^	CI	QTL	Chr.^b^	LOD	Peak	CI^c^	R^2^(%)	Add^d^	Env.^e^
*qPD.A3*	47.82	Bn-A03-p15900189 (47.87)	46.9-49.5	*iq12NJ.A3*	A3	4.48	47.82	46.9-49.5	7.95	6.63	12NJ
*qPD.A5*	31.81	Bn-scaff_18338_1-p345782 (31.83)	27.6-34.0	*iq11NJ.A5*	A5	3.48	31.81	27.6-34.0	6.34	-4.51	11NJ
*qPD.A6*	54.8	Bn-A06-p10135140 (54.74)	49.5-55.5	*iq14DL.A6*	A6	2.82	54.8	49.5-55.5	5.08	-3.03	14DL
*qPD.A9-1*	64.06	Bn-A09-p25823689 (63.70)	59.66-74.36	*iq14NJ.A9*	A9	3.38	64.06	59.66-74.36	6.78	-3.69	14NJ
*qPD.A9-2*	76.36	Bn-A09-p29172005 (76.38)	75.97-76.75	*iq13NJ.A9*	A9	3.34	76.46	75.56-78.26	6.05	-3.95	13NJ
				*iq11NJ.A9*	A9	3.55	76.36	75.56-78.26	6.48	-4.52	11NJ
				*iq12NJ.A9-1*	A9	3.98	76.36	75.56-78.26	7.06	-4.83	12NJ
				*iq14DL.A9*	A9	3.26	76.36	75.56-76.46	6.12	-3.39	14DL
*qPD.A9-3*	81.36	Bn-A09-p30010889 (81.41)	79.86-82.96	*iq12NJ.A9-2*	A9	3.96	81.36	79.86-82.96	7.01	-4.81	12NJ
*qPD.C8-1*	11.8	Bn-scaff_17807_1-p364572 (11.79)	9.4-12.6	*iq11NJ.C8-1*	C8	4.63	11.8	9.4-12.6	8.77	-5.22	11NJ
*qPD.C8-2*	17.7	Bn-scaff_18275_1-p1278049 (17.55)	15.69-19.7	*iq11NJ.C8-2*	C8	5.59	17.6	16.7-22.8	10.47	-5.71	11NJ
				*iq12NJ.C8-1*	C8	6.21	17.6	14.7-25.8	11.29	-6.09	12NJ
				*iq14NJ.C8*	C8	3.65	17.6	15.6-26.5	7.11	-3.77	14NJ
				*iq13NJ.C8-1*	C8	5.48	17.9	16.7-26.5	10.24	-5.16	13NJ
				*iq14DL.C8-1*	C8	4.32	17.9	15.5-26.5	8.26	-3.83	14DL
*qPD.C8-3*	27.2	Bn-scaff_17227_1-p700248 (27.17)	26.69-27.72	*iq11NJ.C8-3*	C8	3.17	27.2	26.5-32.3	6.12	-4.42	11NJ
				*iq12NJ.C8-2*	C8	4.25	27.2	26.5-31.9	7.89	-5.12	12NJ
				*iq13NJ.C8-2*	C8	4.45	27.2	26.5-28.0	8.4	-4.7	13NJ
				*iq14DL.C8-2*	C8	3.69	27.2	26.5-28.0	7.06	-3.56	14DL


**DL, Dali; NJ, Nanjing; 11, 12, 13 and 14 indicate the years* 2011, 2012, 2013, and 2014, respectively. ^a^The closest marker and the marker position in the AH map. ^b^Chromosome. ^c^The 2-LOD confidence interval of QTLs. ^d^Additive effects. ^e^The experiment in which the QTLs were detected.*

A total of 19 identified QTLs distributed across A3 (1 QTL), A5 (1 QTL), A6 (1 QTL), A9 (6 QTLs), and C8 (10 QTLs) chromosomes were detected in the present study (**Table 3**). These QTLs have additive effects ranging from -6.09 to 6.63, and singly explaining 5.08%–11.29% of the estimated phenotypic variation (PV). The number of identified QTLs detected in different experiments was quite different, ranging from two (14NJ) to five (11NJ). Among them, up to 10 identified QTLs were distributed on C8, implying that the major genes for PDgr might exist on the C8 chromosome. Eighteen of the 19 identified QTLs had negative additive effects, suggesting that the normally petalled parent ‘Holly’ contributed favorable alleles for increasing PDgr. Only one identified QTL, *iq12NJ.A3*, had the positive additive effect of 6.63, indicating that the positive alleles for higher phenotypic values were inherited from the apetalous parent ‘APL01’.

**FIGURE 4:**
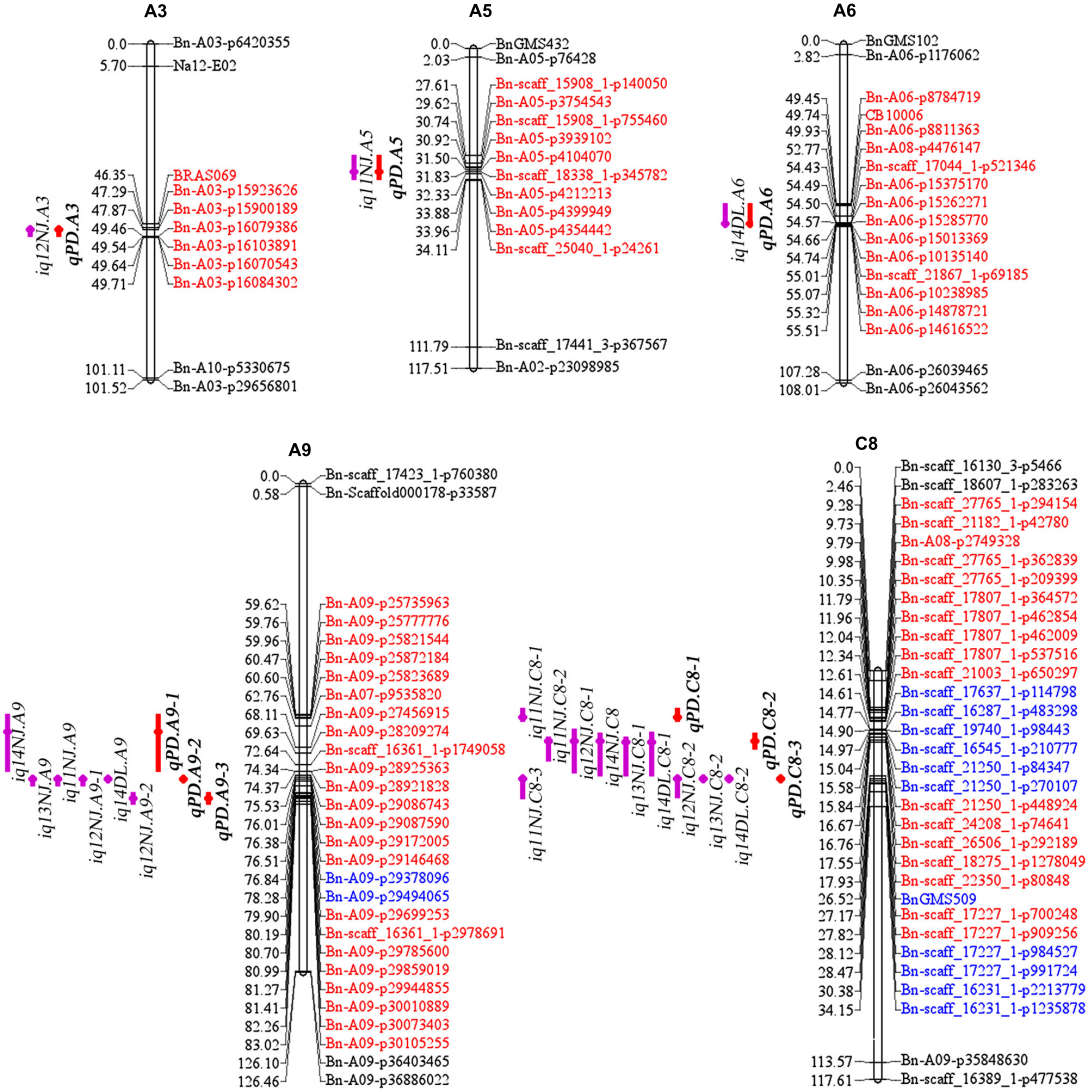
**The locations of identified and consensus QTLs associated with PDgr in the AH map.** The linkage groups are represented by vertical bars. The loci names are listed on the right side of the linkage groups, while the loci positions are shown on the left side of the linkage groups. For simplicity, only the markers underlying the QTL CIs and the terminal two markers of each linkage group are shown. QTLs for PDgr are represented by bars with different backgrounds on the left side of each linkage group (purple bars, identified QTLs and red bars, consensus QTLs). Red loci are underlying the CIs of consensus QTLs and blue loci are underlying the CIs of identified QTLs but not consensus QTLs.

There were 13 identified QTLs with overlapping CIs, and these were further integrated into three consensus QTLs using a meta-analysis method (**Table 3**; **Figure 4**). As a result, the average CIs of these QTLs were reduced from 5.55 to 1.94 cM, which significantly increased the accuracy of the estimated positions of the meta-QTL. The other six non-overlapping QTLs were also considered as consensus QTLs. In total, nine consensus QTLs for PDgr were obtained in the present study. Among these consensus QTLs, one QTL (*qPD.C8-2*) with the closely linked marker Bn-scaff_18275_1-p1278049 of 0.15 cM, was consistently detected in all of the five experiments and had additive values in the range of –0.69 to –3.77 (**Table 3**). According to the description of Shi et al. (2009), if a consensus QTL presents at least once with PV ≥ 20% or at least twice with PV ≥ 10%, then the QTL can be regarded as a major QTL. The QTL *qPD.C8-2* with PV ≥ 10% in 11NJ, 12NJ and 13NJ (10.47, 11.29, and 10.24%, respectively) was the major QTL, which might harbor major genes responsible for PDgr. In addition, two QTLs, *qPD.A9-2* and *qPD.C8-3*, were expressed steadily in four experiments, with linked markers of Bn-A09-p29172005 and Bn-scaff_17227_1-p700248 and explained 6.05%–7.06% and 6.12%–8.40% of PV, respectively. The genes associated with these QTLs controlling PDgr may be less affected by environment, which is consistent with the results of the genetic analysis, which indicated that PDgr in the AH population has a high heritability (**Table 1**). A striking finding was that, except for the three abovementioned QTLs, no other QTL was detected in multiple experiments, and the remaining six QTLs were environment-specific QTLs detected only in one environment (**Table 3**).

### Identification of Candidate Genes Responsible for PDgr in the AH Population

As mentioned above, *qPD.A9-2*, *qPD.C8-2*, and *qPD.C8-3* were the three steady QTLs that showed significant effects in four, five, and four environments, respectively (**Table 3**). The three QTLs span regions of 75.97–76.75 cM, 15.69–19.7 cM, and 26.69–27.72 cM, with physical regions of 0.35, 2.65, and 0.26 Mb on chromosomes A9, C8, and C8, respectively. Based on the comparative mapping between the AH map and the *B. napus* reference genome, 58, 220, and 50 genes, respectively, were identified underlying the CIs of the three QTLs (Supplementary Table S2A). The other six consensus QTLs were only detected in certain geographical regions, suggesting that they were easily influenced by the environment, and genes underlying these QTLs were not analyzed in the present study.

**FIGURE 5:**
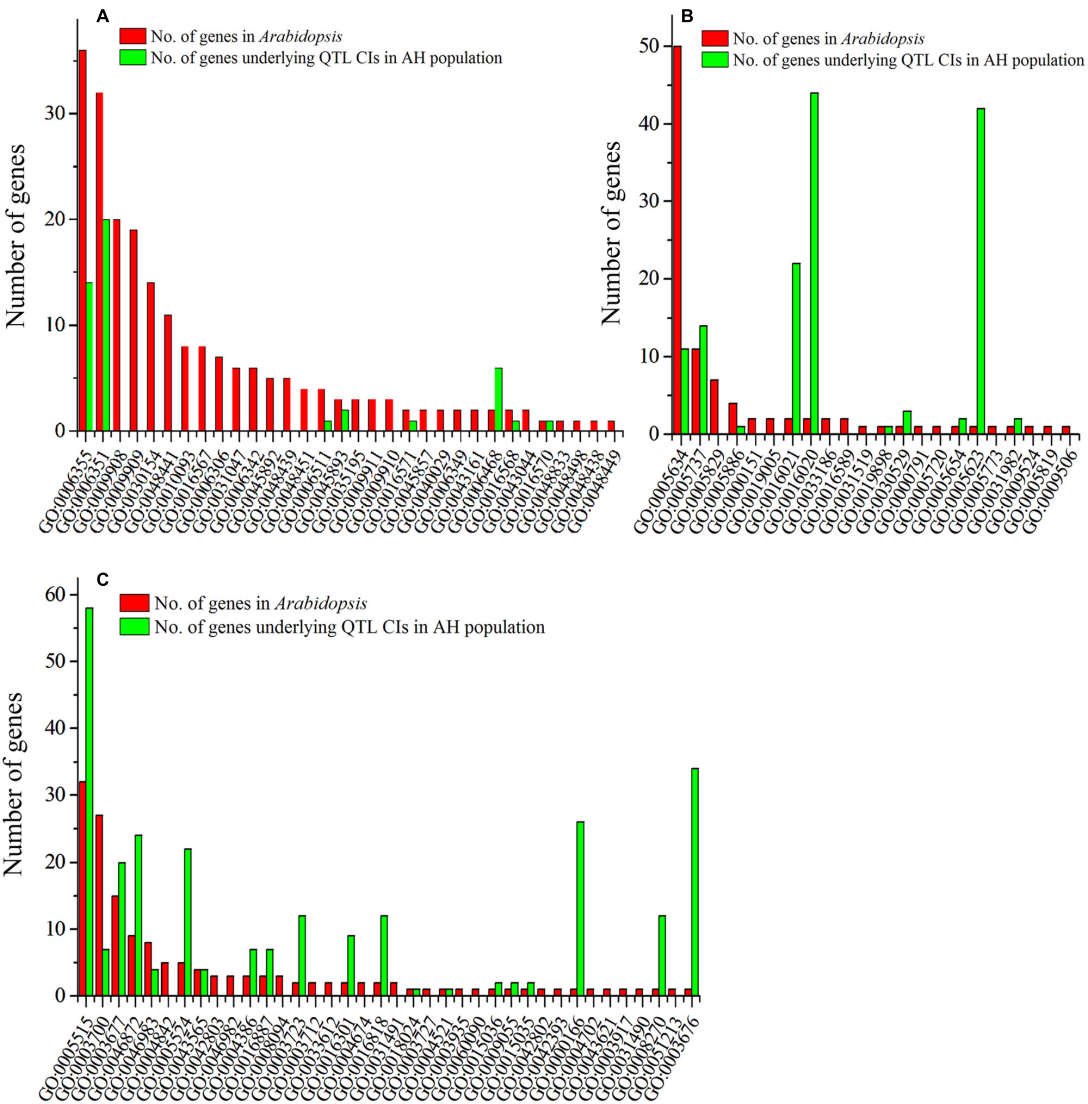
**Gene ontology (GO) annotation of collected genes regulating petal development and the candidate genes of three steady QTLs.** The x-axis represents the functional groups (GO accessions), and the y-axis represents the number of genes in each category. **(A)** Biological process; **(B)** cellular component; and **(C)** molecular function.

Fifty-two genes regulating floral development were collected from 55 published studies that have been performed mainly in *Arabidopsis* (Supplementary Table S2B). These genes were further annotated with gene ontology (GO) terms, which were classified into three categories: biological process (BP, **Figure 5A**), cellular component (CC, **Figure 5B**), and molecular function (MF, **Figure 5C**). The 52 genes were classified into 92 functional groups, with the number of genes in each GO term ranging from 1 to 50 (Supplementary Table S2C). In the BP category, the most abundant GO terms were GO:0006355 (regulation of transcription, DNA-templated) and GO:0006351 (transcription, DNA-templated). In the CC category, GO:0005634 (nucleus) was the most abundant, followed by GO:0005737 (cytoplasm). In the MF category, GO:0005515 (protein binding) and GO:0003700 (sequence-specific DNA binding transcription factor activity) were the major GO terms.

Gene ontology assignments were also used to classify the functions of the 328 genes that underlie the three steady QTLs. The results showed that 146 genes were distributed under 38 of the 92 GO terms (**Figure 5**, Supplementary Table S2C), and thus are potential candidate genes for PDgr in *B. napus*. Among these, 34 (14 and 20, respectively), 25 (11 and 14, respectively), and 64 (57 and 7, respectively) genes were classified in the top two GO terms in the BP, CC and MF categories to which the published genes for petal development were assigned, respectively (**Figure 5**, Supplementary Table S2C). Some candidate genes were assigned to more than one category. For example, *BnaC08g10960D* and *BnaC08g14030D*, which underlie the CIs of *qPD.C8-2* and *qPD.C8-3*, respectively, were assigned to all three categories. In addition, seven, eight and five candidate genes were simultaneously assigned to BP and CC, BP and MF, CC and MF categories, respectively.

## Discussion

The apetalous genotype is a morphological ideotype for increasing seed yield compared with the fully petalled variety in *B. napus*, which improves light penetration through the floral canopy and lowers the incidence of *S. sclerotiorum* infection (Mendham et al., 1981, 1991; Jamaux and Spire, 1999; Jiang, 2007). Having suitable parental genotypes is critical for identifying the inheritance and the genetic bases of a character; however, the lack of completely apetalous genetic resources has been a limiting factor for understanding its genetic control in *B. napus*. Several apetalous flowers in *B. napus* were used to investigate the genetic models for the apetalous character (Buzza, 1983; Lu and Fu, 1990; Kelly et al., 1995; Zhao and Wang, 2004; Chen et al., 2006a; Zhang et al., 2007b,c). In the present study, an apetalous line ‘APL01’ and a normally petalled variety ‘Holly’ were used to construct the AH RIL population and perform a genetic analysis of the apetalous trait. The results showed that PDgr in *B. napus* was controlled by the combination of two major genes and polygenes, which was consistent with previous studies (Buzza, 1983; Kelly et al., 1995; Jiang and Becker, 2003; Chen et al., 2006a; Zhang et al., 2007b). Other genetic models for apetalous in *B. napus* have also been reported, such as the apetalous character being controlled by only one gene locus (Zhao and Wang, 2004), and four pairs of recessive genes (Lu and Fu, 1990; Zhang et al., 2007c). These studies demonstrated that PDgr in *B. napus* is a quantitative trait.

Quantitative trait loci mapping is an effective approach to dissect the genetic mechanisms of quantitative traits, while high-density map can increase the precision of QTL localization and effects, especially for small and medium sized QTLs (Almeida et al., 2013; Stange et al., 2013). However, linkage mapping studies in *B. napus* were mostly based on low-density genetic maps constructed using SSR markers, or higher-density maps based on anonymous markers, such as amplified fragment length polymorphisms or sequence-related amplified polymorphisms (Sun et al., 2007). In the present study, a high-density SNP map was constructed with 2,812 loci involving 11,458 SNPs, covering a length of 2,027.53 cM and with an average marker interval of 0.72 cM, suggesting that it might be extremely useful in QTL detection. Compared with the previously published genetic maps constructed using SNPs in *B. napus* (Liu et al., 2013; Cai et al., 2014; Zhang et al., 2014), the AH map was one of the highest density maps with average distance between loci of less than 1.0 cM. In addition, the genome sequence of *B. napus* has been released (Chalhoub et al., 2014), which will facilitate the fine mapping of QTLs for quantitative traits and marker-assisted selection breeding in *B. napus*. A high-density SNP map is beneficial for the accurate alignment of the AH map to the physical chromosome segments in the assembled *B. napus* genomes. The results showed that the AH SNP map had good collinearity with the *B. napus* reference genome, indicating the high quality and accuracy of the map (**Figure 3**). Furthermore, the A sub-genome of the AH map showed a near-complete coverage of the *B. napus* genome (93.72%), considerably higher than the C sub-genome (76.91%; **Table 2**). One possible reason for the low coverage of the C sub-genome is that a form of distorted segregation in some chromosomes caused by rearrangements among homologous chromosomes in the parents occurred (Lu et al., 2013), resulting in the low coverage of C1 (33.45%), C3 (43.70%), and C5 (34.17%). The loci density in the A sub-genome (0.61 cM/marker) was also higher than that of the C sub-genome (0.88 cM/marker) in the AH map (**Table 2**), indicating the higher polymorphism rate in the A sub-genome. In agreement with these findings, Liu et al. (2013) constructed a map including 976 loci in the C genome and 1,819 loci in the A genome, with an average distance between markers of 0.53 cM in the A genome and 0.93 cM in the C genome.

A large population, a high-density genetic map and replicated experiments in multiple environments are three necessary factors for precise QTL detection (Yu et al., 2011; Wang et al., 2013). In the current study, a QTL analysis for PDgr was based on the high-density AH map and phenotypic data from five experiments, and nine consensus QTLs were identified on the A3, A5, A6, A9, and C8 chromosomes. Until now, only a few studies focused on the complex genetic mechanism of PDgr in *B. napus*. Several molecular markers associated with the petal-loss trait were located on A4 (Fray et al., 1997; Chen et al., 2006b) and C4 (Fray et al., 1997), and four QTLs for PDgr were located on A5, A6, A8, and C5 (Zhang et al., 2007a). These results suggest that the seven QTLs on A3, A9, and C8 in the present study were potential new QTLs, including the major QTL *qPD.C8-2* detected in all of the five experiments and two steadily expressed QTLs (*qPD.A9-2* and *qPD.C8-3*) identified in four experiments. The environment-specific QTLs *qPD.A5* on A5 and *qPD.A6* on A6 were not confirmed due to the lack of common markers between the different populations. The number of QTLs for PDgr in this study may be underestimated because the density of the AH genetic map was not saturated (**Table 2**), which may result in an incomplete resolution of QTLs. On the other hand, as AH population has been tested for 4 years in NJ, QTLs in DL might be underestimated based on the data of one year, and QTL detection could be improved in multiple experiments in DL location. These findings advanced our understanding of the genetic control of PDgr regulation in *B. napus*, and the three stable QTLs are helpful for fine mapping and cloning of QTLs, and will enable a marker-accelerated backcrossing programs.

A typical flower consists of four different types of organs arranged in four whorls, with sepals, petals, stamens and carpels in the outermost to innermost whorls. According to the ABC model, combinatorial interactions between the three classes of floral homeotic genes are affected in the four floral organs, with ‘A’, ‘A+B’, ‘B+C’, and ‘C’ specifying sepals, petals, stamens and carpels, respectively (Coen and Meyerowitz, 1991; Honma and Goto, 2001; Theißen and Saedler, 2001). The isolation of novel floral mutants in *Arabidopsis* and other species has led to an expansion of the ABC model to include the ‘D’ and ‘E’ functions. Thus, the ABCE model was proposed, which established that the organ-specific genes required the activity of *SEPALLATA* genes (Pinyopich et al., 2003; Krizek and Fletcher, 2005). These genes (termed class E genes), together with the class B and C genes, are required for the specification of organ identity in the petal (‘A+B+E’), stamen (‘B+C+E’), and carpel (‘C+E’) (Theißen and Saedler, 2001). Mutants with defects in the second and third whorls (‘B’ function) result in the homeotic conversion of petals to sepals and stamens to carpels (Causier et al., 2010). However, the apetalous line ‘APL01’ used in the present study had normal sepals, stamens and carpels, suggesting a more complex molecular mechanism of floral development in the allotetraploid species *B. napus*. In the present study, the genetic basis of floral development in *B. napus* was analyzed at the QTL level. Three QTLs (*qPD.A9-2*, *qPD.C8-2*, and *qPD.C8-3*) were stable across multiple environments, suggested that the genes controlling PDgr might underlying the CIs of the three QTLs. *B. napus* has a common ancestor with *Arabidopsis*, and a high degree of sequence similarities and chromosomal colinearities are expected because of the progenitors diverged about 20 million years ago (Yang et al., 1999; Koch et al., 2000). Generally, the allotetraploid genomes *B. napus* may typically contain six distinct alleles for each gene present within *Arabidopsis* (Lysak et al., 2005), with the likelihood that genes carry out the core biological processes will be probable orthologs. Based on the GO assignments, 52 genes regulating of floral development mainly in *Arabidopsis* were classified into 92 functional groups, and 146 genes underlying the CIs of the three QTLs were distributed under 38 of the 92 GO terms. These genes were considered as potential candidate genes responsible for PDgr in the AH population. The B-class genes, represented in *Arabidopsis* by the MADS-box genes *APETALA3* (*AP3*) and *PISTILLATA* (*PI*), experienced gene duplication events (Kramer and Hall, 2005). In *B. napus*, two types of *AP3* genes, *B.AP3.a* and *B.AP3.b*, share a high similarity in amino acid sequences, except for an eight residue difference located at the C-terminus, and, the *B.AP3.a* specified petal and stamen development and *B.AP3.b* only specified stamen development (Zhang et al., 2011). Surprisingly, no A, B, C or E class genes from the ABCE model were identified underlying the three stable QTL CIs, indicating that novel genes for PDgr in *B. napus* might exist. To gain a better understanding of how the three QTLs control PDgr in rapeseed, it is necessary to isolate these loci through a map-based cloning strategy in the future study. This study provided useful information for understanding the genetic control of floral development in *B. napus.*

## Author Contributions

XW and KY carried out the QTL analysis and wrote the manuscript. HL, QP, FC, and WZ participated in the field experiment. SC and MH made helpful suggestions to the manuscript. JZ designed, led, and coordinated the overall study.

## Conflict of Interest Statement

The authors declare that the research was conducted in the absence of any commercial or financial relationships that could be construed as a potential conflict of interest.
